# On the Treatment of Pulmonary Consumption, &c.

**Published:** 1845-01

**Authors:** 


### ART. XV.

1. *Du Traitement de la Phthisie Pulmonaire. Quelques reflexions sur les Phthisiques observés à l'Hôpital Saint-André de Bordeaux.* Par EMILE L. PEREYRA, Médecin titulaire du dit Hôpital.—*Paris and Bordeaux*, 1843.

*On the Treatment of Pulmonary Consumption, &c.* By EMILE L. PEREYRA, Physician to the Hospital St. André Bordeaux.—*Paris and Bordeaux*, 1843. 8vo, pp. 84.

2. *Observations on the Treatment of some forms of Incipient Phthisis.* By JOSEPH BELL.—*Glasgow*, 1844. 8vo, pp. 22.

3. *A Practical Inquiry into the value of Medicinal Naphtha in Tubercular Phthisis.* By E. O. HOCKEN, M.D. &c —*London*, 1844. 8vo, pp. 72.

IS CONSUMPTION CURABLE? We are far from saying that it is not so. No philosophical physician, looking at the history of his science and art, can be justified in so believing. It is, however, a melancholy fact, of which evidence need scarcely be adduced, that no plan of treatment hitherto proposed can boast of even the humblest success in permanently arresting the progress of this dreadful malady. Nevertheless, we must not, and we do not despair. Things seemingly desperate have been achieved ere now even in medicine. We ourselves " 'bate no jot of heart or hope," that the great deed, still to do, will yet be done. That we might not, in our little sphere, obstruct even any possible advance towards this goal of the desires of all good men, we have ever been most anxious to keep inquirers in the right path. The false fires which in all times have surrounded it—whether set up by honest ignorance or kindled by the unhallowed hands of empiricism for its own selfish ends—have had the same baneful influence on the progress and prospects of the true therapeutics of consumption: and if we have heretofore raised our voice against the preconization of individual drugs and partial appliances, in its treatment, it is because the principles on which the practice was based were unsound or visionary, and the evidence on which the alleged proofs of benefits rested, utterly valueless. Men ignorant, not merely of the natural history, pathology, and diagnosis of this particular disease, but ignorant of the common phenomena of living bodies, of the fundamental principles of therapeutics, and of all the laws that govern evidence in matters of science, can hardly be deemed capable of even searching for a remedy, much less can they be supposed qualified to find one, or to recognize it when found. It is at once melancholy and humiliating to review the enormous farrago of drugs and agents of different kinds, brought forward, within the last few years, as more or less infallible *cures* of phthisis; brought forward, too, for the most part, by men within the pale of the profession; and then to reflect on the present position of these remedies in the estimation of all rational and experienced practitioners.* Should not this consideration alone teach us that the propounders of such remedies were all on the wrong track? And does it not justify us in regarding with distrust and doubt, at least, all new propositions of like things by like men? Nevertheless, we feel ourselves bound, in defiance of augury and regardless of probabilities, to let no proposition of the kind pass without examination. On this principle we set about our present task, with honesty and good faith, at least, if with but little alacrity and with still less confidence of arriving at a gratifying result.

I. M. Emile Péreyra having, by a series of reflections, convinced himself of the identity of tuberculous and scrofulous diseases, and having heard, and also perused in the medical journals, sundry narratives of the wondrous efficacy of cod-liver oil in the latter affections, "*commenced, in* 1838, *to swerve from the path of death in which medicine was then walking.*" The meaning of this pompous announcement is, that M. Emile Péreyra began, in the year of grace mentioned, to prescribe cod-liver oil to such phthisical persons as fell under his care. And the result of the author's departure from the beaten track must at least have been excessively gratifying to himself; and not a little lucrative, too, if private patients were rescued from destruction as readily as the inmates of the Bordeaux Hospital: for such has been his success, that, in 1843, "the period had arrived when, conscientiously speaking, it would have appeared to him *a criminal neglect of the interests of humanity*, not to make that success known by publication." The *brochure* before

* The following list contains onty a portion of the remedies alluded to: Chlorine inhalations; iodine inhalations; mercury; hydrocyanic acid; creasote; iodide of iron; digitalis; sal ammoniac; carbonate of potassa; common salt; chloride of lime; inhalations of the vapour of tar; the production of emphysema by a system of forced respiration; dry cupping or traction; liquor potassæ; the application of a seton; daily vomiting under the influence of emetics; iodide of potass and sarsaparilla; cod liver oil; wet linen rags to the chest; mechanical extraction of the tuberculous matter through the walls of the chest, &c. &c.

us is the result of this conscientious feeling: we proceed to examine what reasons its pages afford for regretting that the author did not even earlier proclaim the powers of cod-liver oil in curing or arresting the progress of phthisis.

Cases are of course given, to substantiate the general statements made. The day has come, when the veriest charlatan (with which class, be it fully understood, we seriously believe the author is wholly unconnected) feels that some evidence is indispensable to produce any effect.

CASE I is that of a young man, having an enormous cavity in the upper lobe of the right lung; "the frequency of the pulse, the cough, the puriform expectoration, the copious night-sweats, the emaciation, allowed of no doubt that the disease *must* promptly terminate by death." But cod-liver oil and nourishing diet restored the patient's flesh and gaiety, and "in two months he left the hospital in a very satisfactory state." He had a seton under the right clavicle. The simple reflections which this case suggests, admitting the description given to be correct, is, that the symptoms enumerated do not by any means prove that death *must* have followed soon; and that not a few consumptive persons of the lower classes will undergo quite as marked improvement under the use of the very simplest medications, aided with nutritious diet, and strict attention to the general health.

CASE II. A prostitute, aged 18, had tubercles in great quantities, and "*two* cavities, of some size, at the upper part of the right side." How M. Péreyra distinguished the *two* cavities, he does not inform us: a neat piece of diagnosis, however, which would have been worth making public. Frequent pulse, loss of appetite, emaciation, hectic fever, cough, purulent expectoration, night-sweats, and suppression of catamenia, made the case, at the end of five months' illness—unquestionably a very serious one. A seton was placed over *one* of the cavities, the patient was nutritiously fed, and given two tablespoonfuls of the oil daily. In two months, at the outside, this girl left the hospital (March 1839), menstruating again, fat, strong, gay, exhibiting colour in her cheeks, and, above all, according to the nice diagnosis of her physician, with her "crude tubercles somewhat smaller;" the cavities, more refractory, continued there still. Thanks to the cod-liver oil, this patient was enabled to delight the youth of Bordeaux, by a resumption of her official duties in the streets; over-exertion, coupled with a want of due care in the distribution of her favours, led subsequently to her occupying a bed in the Venereal Hospital, and to the painful necessity of swallowing some "nasty blue pills;" but all this had done her lungs no sort of harm, when, in June 1842, she again fell under the observation of M. Péreyra, with an attack of "slight bronchitis." Ausculted at this period the chest furnished pectoriloquous resonance in the same place as before; "but the cavities appeared much smaller than before; there was no *tuberculous noise*, consequently there were no crude tubercles; and the respiration was natural in the remainder of both lungs." This case is put forward as perfectly conclusive, and demonstrating the cure of phthisis, by suspension of the disease and absorption of tubercles. In our minds, it simply proves the resolution of chronic pneumonia, under the influence of attention to diet, and removal froma life of debauchery and misery. The very stress which the author lays upon the phenomenon of pectoriloquy, leads us to doubt his skill as a practical auscultator; the woman is alleged to have had cavities, and to have *expectorated profusely*, yet nothing is said of cavernous rhonchus; the lung of the other side, we are led to conclude, was free from disease, as no statement is made of morbid signs furnished by it. Is it more likely, then, that the right organ had been the seat of pneumonia, or that it contained "two" tuberculous excavations? Any one in the habit of observing phthisis will have no difficulty in answering, infinitely the former. Lastly, after two months' treatment, the "crude tubercles appeared smaller:" imagine such a diminution being effected in two months in the size of tubercles, that auscultation should be able to mark out its extent! Is this likely; or is it not a trifling degree more probable that inflammatory consolidation had been the real condition present, and given way in the manner suggested?

M. Péreyra, however, having some misgivings as to his auscultatory skill being readily admitted, conceives it judicious to relate a case in which death—in spite, alas! of cod-liver oil—gave him the opportunity of showing that he might make a correct diagnosis. A boy aged 9 years laboured under intense fever, constant cough, colliquative sweats, suffocation, &c., and was found, on auscultation, (M. Péreyra, by the way, does not appear to practise percussion,) to have a considerable quantity of crude tubercles and a cavern on the right side, (whether single or double, M. Péreyra's cavities are always on the right side.) This child took the oil for a month, and left the hospital on the 16th of June, almost free from cough, without fever, fat, gay, and with a good appetite. On the 29th of July he was readmitted in an alarming state, (he had not taken the oil in the interim,) and died, in spite of its readministration, on the 14th of July. There *was* a cavity in the right lung, and crude tubercles in both lungs and several other organs. Now, this case, if considered alone, might certainly tend to impress us with a very exalted notion of the powers of cod-liver oil; but when we know, as a matter of certainty, that such temporary suspensions of the disease as that described are observed in cases where NATURE, and nature only, can claim the honour of the fortunate change—when, in a word, it has been proved to demonstration that, out of a given number of cases of phthisis, a certain proportion will stop short, temporarily, at some time or other, no one can say why or wherefore, and no matter what may have been the system of treatment adopted, or whether any treatment at all has been put in force—the efficacy of the nauseous drug sinks vastly in our estimation, and we can find no motive for regarding its administration and the suspension of the disease, in M. Péreyra's case, as otherwise than a simple coincidence.

M. Péreyra employs ferruginous preparations in cases where the symptoms of anemia are present,—a circumstance to which we advert for the purpose of mentioning that the only preparation of iron which the Bordeaux physician has found to be unbearable by phthisical patients, "on account of the irritation it produced, even in very small doses," is the ioduret. The *ioduret!* the very salt of which M. Dupasquier of Lyons, has sung the triumphs, in every conceivable form, as *κατ'εξοχην*, *the* panacea in phthisis.

M. Péreyra places a seton close to the anus, whenever a phthisical patient suffers from habitual pain in that region, loses a few drops of blood *per anum* occasionally, has a hemorrhoid which alters in bulk from time to time, and, in a word, whenever a kind of morbid action appears desirous of establishing itself in that quarter." We have the satisfaction of learning that this miserable infliction upon wretches worn with sufferings of various other kinds, has "appeared to the author productive of good effects."

But the writer is not merely a therapeutist. He aims at acquiring a place among those distinguished for their powers of diagnosis, and commenced with ardour about 1834, to seek "what signs, if any, might be esteemed indicative of the presence of non-ulcerated tubercles." He appears to have kept the results of this goodly determination profoundly secret, until news reached Bordeaux that M. Fournet had announced to the Academy of Medicine of Paris, the facts he had ascertained concerning the early detection of phthisis. The news led to M. Péreyra's reading a paper before the Medical Society of the former town, in which he made known the results of his own inquiries, and these results are as follow. A healthy respiration consists of an inspiratory and an expiratory sound, the former the *shorter* and the louder of the two. In the case of "miliary tubercles," on the contrary, things change; inspiration going forward as usual, expiration terminating abruptly, may be altogether inaudible, and, as a general proposition, only occupies three fourths of its natural time. When miliary tubercles accumulate in greater abundance, inspiration continues free, but the expiratory murmur closes abruptly, and inspiration immediately recommences. A special sound, like that of small metallic bodies rolling on each other at a distance from the ear, is another phenomenon connected with the same anatomical state, or with commencing softening. Such are the ideas which M. Péreyra hastened to communicate to his *chers confrères*, so as to obviate the chance of his being accused of plagiary, when M. Fournet's notions should be made known in detail. What was the astonishment of the ambitious Bordelais on learning that the Parisian had arrived at results diametrically opposed to his own,—at all events his worst enemy could not accuse him of the literary crime from which he was so anxious, even by anticipation, to shield his fair renown. But M. Péreyra, like a good arguer, sticks to his point,—M. Fournet, he maintains to have been led astray by preconceived ideas, &c. Now it is too well known at the present day, that the fundamental principles taught by M. Fournet, (borrowed as they were from Jackson and Louis, and confirmed by a multitude of others also, *before* he chose to adopt them,) are correct, however they be loaded with exaggeration and absurd refinements, to require our pronouncing a verdict against M. Péreyra. The laudable effort of our author should not indeed have been noticed, but from its connexion with his capability of diagnosticating the disease which he is anxious to persuade us he has cured with cod-liver oil.

M. Péreyra conceives he can (as others have imagined also) detect phthisis with certainty, from the characters of the pulse. There is certainly a peculiarity about the pulse of the consumptive, which, taken in connexion with other circumstances, lends its aid in determining the nature of their disease; we confess we can more readily appreciate this peculiarity at the bedside (it is not *mere* threadiness, quickness, and frequency combined, than convey its characters by words. M. Péreyra's test of the phthisical pulse stands in precisely, as it appears to us, the opposite predicament; it is most easily describable, but we fear, from such trials as we have already made, extremely difficult to detect. "The four fingers of the observer's left hand are to be placed upon the radial artery of the right fore-arm of the patient (and vice versâ), in such manner that the index finger rests upon the vessel immediately above the styloid process of the radius, the other three fingers, side by side, pressing the artery sufficiently to feel it distinctly; if, things being thus arranged, the pulse be more particularly felt by the median finger, we have the pectoral or, rather, pulmonary pulse: this exists in bronchitis, pneumonia, pleurisy, tubercles, &c. But where there are sufficient miliary tubercles in the lungs to disturb the general health, there is always a slight jerk, particularly felt by the median finger, immediately before the pulsation. The pulse has in fact something of the *bisferiens* character, but still cannot be confounded with it; the pulsation is a single one, but it seems as if the blood strikes the artery before it circulates onwards; I may make this more intelligible by comparing the divisions of the pulsation to a demisemiquaver (or grace-note) set before a crotchet." Will our readers (who we know must detest bad puns) pardon us for fearing that the whole thing is a crotchet?

M. Péreyra has also succeeded in curing most obstinate cases of true *porrigo favosa* with cod-liver oil.

The work concludes with statistical tables of the phthisical cases which came under the author's treatment. No additional satisfactory evidence appears of the real efficacy of the vaunted oil; and we think it fair to infer from M. Péreyra's own facts, that the specific is destined to take rank with the multitude of other medicines put forward from time to time with similar pretensions—sometimes honestly and ignorantly, generally dishonestly and knowingly—to fret its brief hour on the stage, and then be heard no more of.

II. Dr. J. Bell proposes to confine his observations to the treatment of "some forms of bronchitis and pneumonia occurring in persons of scrofulous constitutions, inflammatory affections which so frequently precede the development of confirmed phthisis, that they may be considered as incipient forms of the disease." It is known that some years past, Dr. O'Beirne, of Dublin, tried mercury in scrofulous inflammation of the joints, with results so favorable, that some of the physicians of the Irish capital thought it would be well to apply the treatment to cases of pulmonary "scrofulous inflammation," which means inflammation of the lungs occurring in individuals having the characters of skin, lips, hair, eyes, &c., which custom assigns to the strumous constitution. It may be remarked *en passant*, that such characters (they are too well-known to need being set down,) are rather rare than otherwise among consumptive people, but this is of course a matter of secondary importance. Be this as it may, the success of this medication was such, that Dr. J. Bell thought he could not act more wisely than in imitating the Irish practice, whenever an opportunity occurred.

He relates seven cases. In the first of these, (as he concludes himself, and as is obvious from his narrative,) the disease was bronchitis of both upper lobes; in the fourth, bronchitis with parenchymatous congestion; in the fifth and sixth, pneumonia; in the seventh, tubercles. The subjects of the first six cases recovered; we can see nothing mavellous in this, people having bronchitis and pneumonia do sometimes recover without (so say the least, Dr. Bell,) having had their gums made sore with mercury, and no shadow of evidence appears that the cure was more rapid, more perfect, or in any way preferable to that which results under modes of treatment of completely different character. The patient in the seventh case not only died, but died in about four months from the occurrence of the first symptoms;—a duration of the disease below the average. The mercury exhibited may, we contest not, be innocent of having hastened the fatal issue; on the other hand, at all events Dr. Bell will have some difficulty in proving that it kept his patient an additional hour above ground, and, to us it appears, the poor man would have had better reason to thank his medical advisers, had his closing hours not been embittered by the miseries of salivation.

Hence it follows, mercury is *the* remedy "in incipient phthisis," because, on the one hand, one tuberculous person, who used it, died sooner than the vast majority of phthisical people, who have never taken a grain of the mineral; and, on the other, because six persons recovered, after taking mercury, from bronchitis or pneumonia! But, says Dr. Bell, these six persons had fair skins, &c., and other signs of scrofula, and therefore *their* pulmonary inflammations were in fact "incipient phthisis," *so frequently* do such inflammations in such persons terminate in the latter malady. Is this a fact in the first place? Is the generation of tubercles traceable to these inflammations in the manner averred? Indubitably if it be so, it is only so in *extremely rare* cases. We are absolutely weary of repeating this, and of referring to the numerical statements of Louis and Grisolle, (the only really sound evidence bearing on the question in existence,) in its proof. The contrary notion is the mere produce of vague diagnosis and *a priori* reasoning.

But granting Dr. J. Bell the reality of the connexion, he ventures upon assuming in the teeth of all sound experience, between the diseases in question, and granting (what is almost as difficult to concede,) that he actually, by exhibiting mercury saved life, or warded off the alleged sequence of these affections in any of his cases of bronchitis and pneumonia, is he thereby justified in affirming that he has cured "*incipient phthisis?*" Let us put a parallel case. Stricture of the urethra, in almost every instance in which it exists, is the result of gonorrhea; stricture of the urethra leads frequently in certain constitutions to elaboration of phosphatic urine and chronic nephritis, or in other cases to chronic pyelitis with distension of the pelvis and infundibula, and more or less complete atrophy of the proper renal structure. Now, in pursuance of his principle, Dr. J. Bell will be fairly entitled, (as soon as he has cured some half dozen young gentlemen gratified by "a subscription from the ladies" in the shape of a blenorrhagia,) to favour the profession with an essay upon "Cases of incipient atrophous destruction of the kidneys, with chronic pyelitis, &c., cured in a fortnight with epsom salts and balsam copaiba."

III. Next on our list stand Dr. Hocken and Naphtha. Dr. Hocken has been for some time known to those whose avocations require them to watch the proceedings of literary persons, as an individual of unquiet temperament, prone to write *de omnibus rebus.* The character cannot be said to conciliate readers of any description, when taking up a new specimen of Dr. Hocken's pamphleteering faculty. Even the mildest-mannered critic feels disposed to look askance at each additional production of a man who, as far as his writings justify an opinion, does not certainly rise above the average of his compeers in mental qualifications, and yet presumes to put himself perpetually forward as a teacher and guide in matters of science and practice, to each of which men of the highest attainments have considered it necessary to devote the exclusive attention of years, before they felt themselves entitled to assume the office of instructors. But it is agreeable to know that any unpleasant feelings of this cast which might crowd into the mind, on scanning the title-page of the pamphlet before us, as well as certain other misgivings connected with the delicate nature of the particular subject treated in it, disappear, like the baseless fabric of a vision, on perusal of the preface. Here abounds testimony,—furnished too by the person best acquainted with Dr. Hocken, namely Dr. Hocken himself,—sufficiently satisfactory as to the author's purity of motives, and unusual fitness to discourse concerning chest diseases. In the first place, as respects Dr. Hocken's disinterestedness and abnegation of self on the one hand, and his divine charity and spirit of universal love on the other, we learn that "the author has put forth the following essay with a sincere desire to benefit *others*, and entirely without the prospect or probability of benefiting *self*." The italics are the author's own, and doubtless meant to signify that the words so printed were not transposed by an error of the press. It might be contended that there was some similarity between this exordium and the affectionate phraseology wherewith Messrs. Goss and Co. urge the claims of the 'Silent Friend,' the 'Ægis of Life,' &c.; but we doubt not the resemblance can only be regarded as accidental and utterly unconnected with any identity of purpose on the parts of Dr. Hocken and the distinguished advertisers. The author next announces the special refinement of his sympathy for "the consumptive,—falling victims, as many of them do, in the very pride of youth and beauty"—from which they that will it, may safely conclude his doors are as open to the sufferers as his heart to the sufferings. Changing the theme, Dr. Hocken expatiates on the "delicacy and accuracy in hearing," and upon the "taught and thoroughly practised ear," required for the investigation of the additional subject on which he has chosen to enlighten the public. But let not the reader despond, let him not doubt the perfect competency of Dr. Hocken, for the gentleman himself informs all whom it may concern, that "he believes his sense of hearing to be naturally excellent," as likewise that he "has studied under some of the best auscultators in the world." Goods recommended so strongly and conscientiously by their owner are surely worthy of a trial—let us look then into the body of Dr. Hocken's work.

First, what does naphtha do, what are the pretensions with which it is put forward? "The proper or medicinal naphtha," the writer avows, "is by far the most valuable remedy hitherto proposed for the *relief* and in some instances *cure* of this distressing malady;" it is not, however, to be regarded "as a *speedy* and certain *cure* in all forms and stages of phthisis." The last clause furnishes a valuable hint to Dr. Hocken's future patients, (we mean, of course, his *dispensary* patients,) not to lose confidence too soon, or give up attendance so long as they have time [or money ?] to spend in improving their health, and swallowing naphtha.

Secondly, what is the naptha which Dr. Hocken patronizes? It is the same as that originally advertised, and which may still be had at many chemists' shops, placarded as that intended for the "cure of consumption." In other words, Dr. Hocken's naphtha is *the* naphtha, and not the other naphtha.

Thirdly, what is the evidence brought forward by Dr. Hocken that consumption is curable by naphtha, such as he has used? (We grant at once, if he pleases, *argumenti gratiâ*, that the value of the medicine should be tested by his own evidence, and that any one who has ventured to say nay, on his particular experience, was the victim of fraudulent chemists who would give the wrong naphtha.) Why a series of cases, in other words of facts: Now facts are well known to be stubborn things. Let us approach such as we are given, then, with becoming gravity.

The total number of cases referred to is twenty-three,* observed during a period of eleven months; and of these, seven (not six, as stated in the text, p. 67,) died; in other words, as nearly as possible, one third of a series of patients, treated on a certain plan, were cut off by the disease in less than the course of a year. Stating the fact in this broad way and avoiding all inquiry into the stage of the disease, or (what is infinitely more important in respect of prognosis,) the degree and manner in which the constitution generally has suffered, does it plead in favour of efficiency of the plan in question? Indubitably it does not: take a number of phthisical patients in all stages of the disease (eschewing, as carefully as Dr. Hocken did, those who might be considered, from the gravity of their state, actually moribund,) palliate their symptoms in the most ordinary way, and that two out of three will continue alive at the end of eleven months, is as certain as any proposition in medical statistics. But the result of Dr. Hocken's treatment actually falls below the average, when the fact is borne in mind that, first, in seven of these cases, according to his own admission, the disease existed in the earliest stage, and in some to an excessively limited extent; and, secondly, that in many of them the constitutional symptoms were far from being severe. Such is the first general inference we deduce from the contents of this pamphlet.

Our second general inference is one which we cannot for a moment apprehend will prove annoying to Dr. Hocken; such is the low estimate at which that gentleman rates his qualifications in the preface. It is this: that Dr. Hocken's work proves him to be perfectly incompetent to undertake the sound examination of a question in therapeutics. And for these reasons: it will be admitted, we presume, even by the patrons of naphtha, that the natural course and progress of a disease, the proportion of persons it kills, and the average time in which it effects their destruction, should be thoroughly known by him who attempts to show that any given system of treatment favorably modifies that natural course. Now Dr. Hocken would appear, from his never stating an opinion concerning such natural course, to be ignorant of its importance as an element in the set-

* Really twenty-two; for one patient (Case xix) was only seen on a first and single occasion.

tlement of therapeutical questions; if so, he is unfit to grapple with these. He may, however, contend, on the evidence of certain passages quoted from different sources, that he was acquainted with the natural tendencies of the disease towards cure or otherwise.* Let it be so; he continues not a whit the fitter guide in the matter. For one or other of two things: either he is unaware that to prove the value of a treatment he must demonstrate that the mortality and suffering caused by any disease are diminished by *that* treatment below the standards obtainable from *other* treatments; or he knows that such comparison would bear against him, and he deliberately abstains from instituting it. He cannot escape from one or other of these alternatives, and we freely accord him his choice—let him take his seat on which horn of the dilemma he pleases, he has shown himself incapable of understanding the character of a therapeutical problem.

Such are the general inferences to which we have been led by the examination of this pamphlet; we admit that they are insufficient to show the inefficacy of naphtha, though they do the incapacity of Dr. Hocken. Turn we then to the cases themselves.

*Series the first.* Here is a collection of seven cases of the disease in an early stage. Before analysing one or two of these we must observe upon the difficulty which attends the critical examination of the narratives of such cases. A set of physical signs are written down which signify the presence of tubercles in an incipient stage. Now the difficulty of satisfactorily ascertaining these signs is familiar to those who are accustomed to see chest affections. Dulness, to a slight amount especially, as Louis testifies, is a condition which must be discovered on repeated occasions and under various modes and manners of percussion, before we are justified in affirming its existence. Slight deviations from the natural condition of the respiratory murmurs are with equal difficulty to be substantiated. Yet when the signs are put down in black and white they signify the same, whether ascertained by a Laennec or an A. B.; and, unless by some collateral evidence proved to have been incorrectly observed, they must be accepted as actually existing conditions. In some instances groups of physical signs have been collected by curers of phthisis from systematic writers, and the prettiest pictures imaginable drawn;—the very perfection of the details betraying the manner in which the whole effect was produced. Our object in referring to these circumstances is to show that the slightest flaw which can be detected in the narrative of physical examinations, has much more importance than might appear on the surface.

Case I is entitled, "Tubercular deposition in the upper and anterior portion of the superior lobe of the right lung. *Complete recovery from the influence of naphtha.*" Here is a man, aged 23, complaining of in-

* A case is transcribed, from an American journal, of supposed phthisis in which recovery took place after the use of simple cough mixtures. What then is the marvel that recovery should follow occasionally when a little naphtha is added to these, unless indeed this substance be actually detrimental. Is not Dr. Hocken in writing down this case in such a book as that before us, doing very much for himself as the sapient Dogberry would have had done for him? He absolutely is, if he believe that the case really was, as its heading promises, one of "spontaneous cure of phthisis." But the truth is that no particle of evidence exists that this particular case was otherwise than one of empyema.

disposition, "hectic symptoms," (what these were we are allowed to divine,) pain behind the sternum and in the right shoulder, with slight cough, accompanied by expectoration of transparent mucus. He had suffered thus for about two or three months; and came of a phthisical father. The physical signs were "slight dulness of the sternal end of the clavicle and of the upper and sternal portion of the subclavicular region of the right side on percussion; morbidly increased resonance of the voice and heart-sounds; murmurs harsh, expiration prolonged." Now this man was probably tuberculous, admitting the signs (taken in connexion with the general symptoms) to have been correctly observed; what then of the naphtha? Ten minims of the liquid were ordered thrice daily in a glass of water, and by the 6th of April the patient had advanced so far as to "complain of much general indisposition and pain in the right shoulder." It does not appear that much had been gained at this period; but "the dulness on percussion was sensibly diminished and the murmurs less harsh,"—a statement rather difficult to reconcile with the previous account of the general and local symptoms. Let that pass, however. Still Dr. Hocken seems to have waxed doubtful of the influence of naphtha, for he now prescribed gentian internally and a *blister* to the chest; and followed up this notion a week later by keeping up "an eruption over the front of the chest with ung. antim. tart." On the 4th, pain and uneasiness were gone, "the cough and shortness of breath (nothing is said about this symptom before; is it now introduced to make things square?) greatly relieved;" little or no expectoration remained. But he continued "to be fatigued and to perspire freely from slight causes, and to be subject to attacks of faintness." The dulness had "entirely disappeared, and the murmurs became soft and normal." He felt well on May 11th, and continued so in September.

As respects the condition of the physical signs, we more than doubt the alleged fact of the heart's sounds having been unduly audible when the amount of dulness on procussion was so slight as stated; probably Dr. Hocken imagined the alleged increase of loudness of the sounds in deference to systematic authors. The man was probably, as we have said, tuberculous; but if the result in *respect of physical signs* be correctly put down, there can be no question in the mind of any human being that the cause of the original dulness was not the tuberculous accumulation itself, but congestion of the adjoining structure temporarily induced by this. That the tuberculous disease itself had disappeared is not shown; for such an account of it as might give rise to local temporary congestion might be altogether inappreciable by physical signs. Again, symptoms of congestion disappear under the use of naphtha, gentian, a blister, and the powerful counter-irritation of tartarized antimony;—and this case is published as exemplifying "complete recovery from *tubercles* under the influence of *naphtha*." This savors either of imbecility of mind, or of impudence to a degree which it is painful to contemplate.

In CASE II we have the history of a man who had had hemoptysis, and the local and general symptoms of tuberculous disease. Flattening of the upper part of the chest, dulness on percussion; harsh respiration; increased vocal resonance, and unnatural clearness of the heart's sounds are all set down in comely order as confirming the diagnosis. The naphtha was commenced with on the 15th of January, and on the 14th of February (!!)

the report is, "states himself to be perfectly well. Dulness had disappeared; murmurs normal; voice equally resonant on both sides." "States himself to be perfectly well." Art thou a follower of Ignatius Loyola, Dr. Hocken, by profession? Or hast thou simply taken a hint from the pages of Eugène Sue's 'Juif Errant,' concerning the art of making a little bit of Jesuitism tell at a pinch? Thou dost not say, Dr. Hocken, that the man *was* well, but that he *stated* himself to be well. Why so have we known men and women, within the last year alone, to the amount of some half dozen," state themselves to be perfectly well," which individuals had nevertheless cavities or softened tubercles in both lungs. Shall we inform fathers and mothers of families of these "*cures*," in a neat pamphlet bearing our address in prominent type, and telling them how we did it all with "vin. ipecac." and mutton-chops? But no matter for the patient's statement;—the physical signs cry aloud for the supremacy of naphtha. In one month all is changed. Unfortunately energetic Dr. Hocken goes a little too far, or in struggling to avoid Charybdis founders on Scylla. He tells us in truth that the voice was "*equally* resonant on both sides," when he had done with the naphtha. Now, as is well known, this vocal similarity on both sides can only exist when the natural resonance has either risen above par on the left side, or fallen below par on the right. On the first supposition there must have been consolidation on the left side, and if so the patient remained uncured; on the second, Dr. Hocken did more than remove the tubercles, he rarified the lung and would eventually, had the naphtha been continued, have produced, heaven knows what amount of shreddiness of the pulmonary substance. On the one supposition, Dr. Hocken either did not know how to tell, or choose to conceal the whole truth; while the other involves an absurdity which even he will probably shrink from acknowledging. Let him once again have his choice of difficulties, whichever he selects the responsibility of, he will probably not rise in the estimation of his brethren.

Such are the first two cases of this series; they are perhaps the *most* favorable of the set to Dr. Hocken's pretensions. The subject of Case V, after some slight improvement, was lost sight of (or at least nothing more is said of her) five months after the treatment was commenced; she was then in precisely the same state, if not worse, than when first seen. And yet in a tabular view of the cases at the end of the book, the "Results," in this instance, are stated to have been "*symptoms much relieved; physical signs improved.*" Case VI is another of "*cure* by *naphtha;*" the plain English of this phrase being that a tuberculous woman underwent much improvement in her local and general state after having taken naphtha, had a blister to the chest, kept up an eruption in the same situation with tartar emetic, and last, though we apprehend not least, spent a period of several weeks on two occasions in the country, her usual place of residence being one of the infectious purlieus of Soho!

In Case VII blisters were frequently applied, a tartar emetic eruption kept up, blood was taken twice by cupping from the nucha, (to relieve headach, it is stated); the woman had compound conium pill, twice daily, occasional doses of pulv. jalap. co., subsequently four grains of pill. galb. co. thrice daily; three minims of acid hydrocyan. dilut. ter die; at a later period a seton was applied to the nucha, still for the headach; shortly after eight minims of liq. op. sed. were added to each dose of medicine; and next a grain of quinine and three of hydr. c. cret. every night with a scruple of pulv. jalap. co. occasionally. After five months of this treatment (she had an affection of the thumb consequent on a burn,) she was sent to Margate with the sounds on percussion alike on both sides, and the murmurs perfectly normal. Now in point of fact the woman, when first seen, had congestion and bronchitis of the apices, (tuberculous, we question not;) there was sibilant rhonchus to prove this. Time, aided by the heterogeneous treatment adopted (especially the counter-irritation) removed this; and the signs consequently improved. But suppose the patient was actually cured of tuberculous disease, why might not the favorable result have been produced by the seton, the prussic acid, the cupping, the opium, the blisters, or the tartar emetic ointment, singly or combined? Why the first two of these agents have actually had their praises sung (as warmly and as honestly as those of naphtha by Dr. Hocken,) as actual *specific cures* for the disease. But whether they be specific or not is of little consequence. It is enough for our purpose that we find a benefit deliberately ascribed to naphtha, of which there is no shadow of evidence to prove that substance really the author; and all this is done with a coolness and an air of innocent conviction that satisfy us Dr. Hocken might possibly, had he chosen the same walk as that worthy, have eclipsed even Robert Macaire.

The writer "now passes on to consider the operation of medicinal naphtha in more advanced cases of phthisis, but still before the occurrence of softening." In the first of these cases we find, that on the 22d of June there were flattening, impeded motion, very marked dulness, bronchial respiration, diffused bronchophony, great increase of clearness of the heart's sounds, mucous rhonchus with a hollow somewhat metallic character, &c. and on the 21st of September there was no dulness on percussion, and the respiratory murmurs sounded exactly alike on both sides. Now this might pass muster, but when Dr. Hocken adds "expiration being audible on both sides and slightly more prolonged than usual,—*somewhere about one third the length of inspiration, instead of about two to ten*," we confess we have misgivings, and the blight of suspicion crosses our minds. Had Dr. Hocken made himself practically acquainted with the relative duration of inspiration to expiration, he would have known that Fournet's ratio of ten to two was one of the errors into which this accomplished auscultator fell; he evidently supposed that that ratio was generally admitted, and endeavours to produce the impression that he had ascertained it himself. Besides, observe the fine drawing; the confidence and ease with which a ratio of three to one is distinguished from one of five to one! Here indeed we recognize the "taught and thoroughly practised ear." But when we turn to case the ninth, we find (as will presently be seen) such further evidence of the powers of Dr. Hocken's "taught and thoroughly practised ear," as leads us to regard his pretension to distinguishing ratios, very much as we should the presumption of a school-boy who, on bringing his first rule-of-three sum to a conclusion, forthwith writes to his mamma concerning his facility in solving quadratic equations.

CASE IX is one of tuberculous disease considerably more serious in character and advanced in progress. The subject of it, a female, had been under treatment about four months, when (on the 24th of September,) she was discharged in a worse general state, (for emaciation was then on the increase, and the tongue covered with aphthæ,) than when first seen. Yet the "results" of this case, given in the table at the end, are "*great relief to general symptoms,* and improvement of physical signs with occasional relapses." But this is not all, there was further news of the case on the 3d of October. And what news? Was the patient then recovered, and did she present an expiratory murmur bearing the proportion of "one to three" to the inspiratory? No. Was she improving then? No. Was she even in *statu quo?* No. She was simply in that condition in which even Dr. Hocken must have failed to detect respiratory murmurs in the ratio of three to one,—she was dead. Dead! What in scarcely more than a little week, after all had been (the table says it,) going on so well, the general symptoms *greatly relieved,* the physical signs *improved*? Aye dead, in spite of all this. And dead, not in consequence of any intercurrent attack in a distant organ, not from a sudden increase in severity of any symptom or symptoms, but simply and naturally from the ordinary progress of the disease; the woman "sank." But, poor woman, there was not an end of her yet; her "*case,*" (as if in mockery of *herself*) must serve to swell pages sufficiently well calculated to entrap the unwary and bamboozle the ignorant,—from the grave she was summoned to bear mute witness to the powers of naphtha, and to aid in enticing sufferers like herself to confide in the honesty and skill of its vendors.

But let us turn to a less painful view of this case. On the 22d of September, we are informed concerning the physical signs as follows: "The sound on percussion alike on both sides, *slightly duller* (under the clavicles) *than the upper part of the mammary region.* Inspiratory murmurs over the dull portions anteriorly on the right side SLIGHTLY HARSHER *than natural; no* expiratory murmur to be heard; a *few moist crackling sounds* heard over a limited space, *beneath the clavicle near the sternum*; voice and heart not excessively resonant." Now this was the state upon which the statement of "improved physical signs" is based, and a very pretty improvement it doubtless signifies, when compared with those described as existing when the patient was first seen. But hear the anatomical state of things to which these "improved signs" (as shown at the post-mortem examination,) responded. "*Left lung.* Pleural surfaces adherent over the whole upper posterior by bands of false membrane, which admitted of being broken down by the finger, and at the apex of the superior lobe very closely by a thick tubercular layer.... .. A *complete cap or hollow cone of tuberculous matter was left adherent at the apex,* when the lung was removed. The lung itself was *a mass of softening and softened tubercles*....the *only sound portion* being over a *small space inferiorly and posteriorly.* The *inferior lobe was softened throughout,* and dripping with pus....There was *much purulent fluid* in the *lower lobe of the lung,* but many of the tubercles were less softened than in the upper lobe. *Right lung.* The *pleural surfaces* were adherent in an *exactly similar manner,* here as *on the left side,* and the removal of the lung left the *same hollow cone of tuberculous matter beneath the clavicle.* There was *a small portion of œdematous, but crepitant and otherwise healthy, lung* situated superiorly and anteriorly. All *other parts of the lung were loaded with softening tubercles,* but free from caverns." All this would have been almost incredible, had we it not in black and white before us; the number and variety of contradictions between the "improved" physical *signs* and the actual physical *state* are almost inconceivable. Now as we are absolved, we presume, from imagining a miracle to have occurred, and the ordinary physical effects of physical causes to have absented themselves, we find ourselves reduced per force to the very painful dilemma of believing either that Dr. Hocken invented the list of "*improved*" signs, or that he is as ignorant as a sucking babe of physical diagnosis. If Dr. Hocken escape with flying colours from this dilemma, we will, for the remaining term of our natural life, venerate him as the—most ingenious of consumption-curers.

CASE XII furnishes us with the naphtha-cure in another of its phases. A woman after a *week's attendance* "does not feel better in her general health; and grows so exhausted from waiting with the other patients, that she feels herself unable to attend;" but she coughed less and slept better. Might not accident or morphia have done as much? No matter; the table furnishes the case as one of "improvement."

CASE XIII is interesting from the intimation it furnishes that the author left town "temporarily" a week after he had commenced giving naphtha to Mary Anne Paget; and that the said Mary Anne, "unwilling to forego the benefits of the naphtha treatment, placed herself under the *private care* of Dr. Hastings." Dr. Hocken could not of course have given her a prescription to be used during his "temporary absence."

But *ohe! jam satis*, in the way of analysis, both for our readers and ourselves. "In certain quarters the employment of naphtha," says Dr. Hocken, "has been regarded merely as a piece of quackery and imposition, and I felt disposed to regard it as such myself, before I convinced myself of its real value." This is a striking sentence; from it we learn totidem verbis, that Dr. Hocken continued for a certain time, (the duration of which is not specified,) to regard the original patron of naphtha as a quack and impostor. This is the unkindest cut of all for poor Dr. Hastings: the malignity of envious reviewers, abashed by his superiority, and confounded by his successes, might be understood,—they might be expected, from simple envy, to stigmatize him as of doubtful honesty; but "mine own familiar friend, in whom I trusted," who knows me thoroughly, who has watched my manœuvres with that tender anxiety for my interests, characteristic of a dispensary colleague, and who now seeks to fatten on my ideas!—he to assail me with the venom of vituperation! Console thyself, Dr. Hastings; Dr. Hocken will now make up for past delinquencies, for has he not joined in partnership with thee for better for worse, and as we know on the one hand that "birds of a feather, &c.," and on the other we learn, from Dr. Hocken's preface what must, on the principle of that wise saw, be the character of him with whom its author associates, we can predict justice will be done thee in the next pamphlet, as a man that careth not for self, but overfloweth with the spirit of universal love,—that hateth quackery, and proveth his devotion to pure science by—pouring medicinal naphtha over fragments of tubercle on the object-glass of a microscope!

Probably the first clause of the sentence just quoted is levelled at ourselves among others. Under this apprehension, we may as well observe that neither we, nor any honest individual, could in the present state of knowledge object to the "*employment* of naphtha," or regard such employment as quackery. But we do object to, and stigmatize as the quintessence of quackery, the publication of books, in which truth is utterly perverted, and in which, with views that may without difficulty be conceived, naphtha is made to appear capable of producing results which no iota of evidence corroborates.

Of a piece with this cunning version of the real grounds on which our detestation of such books as the present rests, is the insinuation that regular practitioners regarded consumption as a disease necessarily running a steady and unchanging course to death. Here again is an utterly false and perverted view of the reality. No, Dr. Hocken, (and you know this as well as we do,) if phthisis did not, as we have always proclaimed it does, *frequently* stop short *temporarily* in its course, *sometimes* undergo suspension for a *lengthened period,* and in *very rare cases* become *permanently* arrested, (all this in the natural course of things,) phthisis would cease to be one of the strongholds of charlatanism. *We* have always encouraged, instead of attempting to repress, honest attempts to improve the treatment of consumption ; but it does not follow from this, as a precise logical induction, that we are called on to hold out the right hand of fellowship to Hastings, Hocken, and Co.

We are tempted to believe, or perhaps rather to hope, that Dr. Hocken has been betrayed to the production of this pamphlet, less by a deliberate desire to mislead, than by a restless spirit of vanity, and craving for notoriety. The latter indeed seems to be with him a passion. Four pages are stitched into the work, filled with every little scrap of commendation or simply courteous mention, gathered from every possible source, whether distinguished or obscure, that has been bestowed upon any one of Dr. Hocken's literary productions. His success in this direction proves that he would in all likelihood be an excellent person to find a needle in a bundle of straw, but does not appear to us destined otherwise to raise his reputation.

---

### Art. XVI.

*Vestiges of the Natural History of Creation.—Lond.* 1844. 8vo, pp. 390.

